# Variability in the therapeutic response of Metformin treatment in patients with type 2 diabetes mellitus

**DOI:** 10.12669/pjms.35.1.100

**Published:** 2019

**Authors:** Maryam Rashid, Muhammad Shahzad, Saqib Mahmood, Khurshid Khan

**Affiliations:** 1 Maryam Rashid, Department of Pharmacology, University of Health Sciences, Lahore, Pakistan; 2 Muhammad Shahzad, Department of Pharmacology, University of Health Sciences, Lahore, Pakistan; 3 Saqib Mahmood, Department of Human Genetics and Molecular Biology, University of Health Sciences, Lahore, Pakistan; 4 Khurshid Khan, Department of Medicine and Endocrinology, Jinnah Hospital, Lahore, Pakistan

**Keywords:** Type-2 Diabetes Mellitus, Metformin, Glycemic Response, Body Mass Index, GIT Intolerance

## Abstract

**Objective:**

To assess the glycemic response of metformin in patients with Type-2 Diabetes Mellitus (T2DM) as well as to see its association with reductions in BMI and GIT intolerance.

**Methods:**

This Quasi, Experimental study was conducted at Jinnah-Allama Iqbal Institute of Diabetes and Endocrinology (JAIDE) Jinnah Hospital, Lahore from 1^st^ March 2016 to 30^th^ September 2016. Newly diagnosed T2DM patients were given metformin for duration of three months and later on they were categorized into Responders and Non-Responders on the basis of HbA1c (A1C) reductions, which were estimated by Hemoglobin (A1C) analyzer (TD4611A TAIDoc Tech. Taiwan) through photometry. Similarly, baseline BMI and BMI after three months therapy with metformin was also recorded.

**Results:**

Among total of 200 patients, 40.5% of the patients were classified as Non-Responders whereas; 59.5% of the patients as Responders. The baseline BMI (26.09 kg/m^2^) was also decreased significantly after metformin therapy (25.40 kg/m^2^). It was found that metformin reduced the A1C in all the patients. However, the glycemic control was much better in patients with higher baseline A1C (1.13% ± 0.08) as compared to lower baseline levels (0.61% ± 0.07). Regarding GIT intolerance, 140 patients lacked the symptoms, out of which 60.7% were responders and 39.3% were non-responders.

**Conclusions:**

Metformin lead to improvement in glycemic control in 59.5% of newly diagnosed T2DM patients after taking metformin for three months but in 40.5% it did not which may be because of combined effects of various gene polymorphisms and their interaction with non-genetic factors. Metformin reduced the BMI in all the patients; however, BMI lowering activity of metformin was same regardless of its effect on HbA1C. Moreover, the signs and symptoms of GIT intolerance did not differ between the two groups.

## INTRODUCTION

Type-2 diabetes mellitus (T2DM) is the predominant form of diabetes and accounts for at least 90% of all cases of diabetes mellitus.^1^ It is characterized by the insulin resistance due to reduced sensitivity of insulin in the body tissues along with decreased insulin production. Insulin receptors become inefficient in receiving insulin and providing it to the body tissues ultimately accumulating the glucose in blood and in various parts of body.^2^

Worldwide prevalence of diabetes in adults was estimated to be 4.0% in 1995 and is expected to rise to 5.4% by the year 2025. Pakistan will have an escalation from 4.3 million in 1995 to an anticipated 14.5 million in the year 2025.^3^ As per International Diabetes Federation (IDF) report 2015, in Pakistan, 7 million people are diabetics.^4^ The mean prevalence of type 2 diabetes mellitus in Pakistan was previously 11.77% which has now increased to 26.3%.^5^,^6^

Metformin is extensively used for treating T2DM as first-line monotherapy. In conjunction with diet, metformin reduces fasting glucose concentration by 2.78 to 3.90mmol/L (50 to 70mg/dL), which corresponds to 1.3% to 2.0% reduction in HbA1c values.^7^,^8^ Additionally, it is known to prevent or delay the onset of T2DM in those with pre-diabetes. Its major mode of action is to reduce hepatic glucose production, which is increased at least two folds in patients with T2DM. All these effects finally lead to increase in blood glucose levels in diabetic patients.^9^

Metformin is associated with weight loss when used to treat diabetes and thus differs from a number of other anti-diabetic medications that are associated with weight stability or gain.^10^ Metformin treatment is frequently associated with GI side-effects (20-30% of patients). Common metformin GI symptoms include nausea, diarrhea, vomiting, bloating, dyspepsia, metallic taste, abdominal pain, abdominal cramps and/or changes in intestinal motility, leading to loose stools and overt diarrhea that becomes uncontrollable sometimes. The pathophysiology of metformin induced GI intolerance is unclear, however it is hypothesized that GI intolerance is related to high concentration of metformin in the intestine after oral administration of the drug.^11^

The current study was conducted to determine the efficacy of metformin in lowering HbA1C. The decrease in HbA1C could be considered as a criterion for response to metformin. It highlights the need for personalized medications to maintain tight glycemic control.

## METHODS

This Quasi, Experimental study was conducted in Jinnah-Allama Iqbal Institute of Diabetes and Endocrinology (JAIDE) Jinnah Hospital, Lahore from 1^st^ March 2016 to 30^th^ May 2016. Two hundred (200) newly diagnosed patients with T2DM (age 35 to 60 years, metformin naive patients with HbA1C 7-9%) were recruited in the study. Type2 diabetic patients were diagnosed on one of the following criteria; Fasting (8 hour or longer fast) glucose ≥126 mg/dl (≥ 7.0 mmol/liter), Two hours glucose ≥200 mg/dl (≥11.1 mmol/liter) during an oral glucose tolerance test (OGTT), Non-fasting plasma glucose > 200 mg/dl or HbA1c≥ 6.5%. Patients having abnormal renal functions (raised creatinine levels ≥ 1.5mg/dl in males and ≥ 1.4mg/dl in females), cirrhosis of liver, congestive heart failure, pregnancy, peptic ulcer disease and inflammatory bowel disease were excluded from the study. The sample size was calculated by WHO software based on S.K Lwanga and Lameshow. The following formula has been used, keeping the confidence (CI) level equal to 95% and the margin of error equal to 6%.


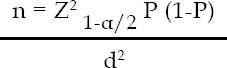


Z^2^_1- α /2_= for 95% confidence level = 1.96

**P** = prevalence = 57.4%

**d** = Margin of error = 6%

**n** = Sample Size = 260

Initially a total of 260 type 2 diabetic patients were included in the study. However, because of inclusion criteria and later stage drop out of patients, the sample size was reduced to 200 in each group.

All the participants provided written informed consent and subjects were adequately informed of the aims, methods, sources of funding, any possible conflicts of interest, institutional affiliations of the researcher, the anticipated benefits and potential risks of the study and the discomfort it may entail and post-study provisions. These protocols were approved by the Ethics Committee of University of Health Sciences, Lahore. Additionally, the research and recruitment protocols were carried out according to the Ethical Principles for Medical Research involving Human Subjects adopted in the Declaration of Helsinki by the World Medical Association.^12^ Information obtained in the interview was recorded on standardized data collection forms. Convenient sampling was done for recruitment of patients.

Metformin was given starting with low dose of 500mg daily and then upgraded to full dose (2000 mg/day) with following schedule: 500 mg once a day for 5 days followed by 1000 mg once a day for five days and finally 1000 mg twice daily, if no side effects were observed. Patients were monitored for a 12-week period. Patients were asked to come for follow up after six week to see compliances.

Blood sampling for A1C estimation was done twice in the study period, first at the beginning of the metformin therapy and the second one; three months after metformin therapy. On the basis of A1C reduction by metformin, patients were categorized into Responders and Non-Responders. There is no accepted criterion in the clinical cut-off point to divide patients into Responders and Non-Responders. Thus, we selected the criteria based on our clinical experiences and previous studies as follows: Responders and Non-Responders (patients whose HbA1c levels had decreased by ≥ 0.8% or < 0.8% from the baseline within three months of metformin therapy respectively).^13^,^14^

A1C was measured by Haemoglobin (A1C) analyzer (TD4611A TAIDoc Tech. Taiwan) through photometry. The reagent utilizes antigen-antibody reaction to directly determine the glycated hemoglobin in the blood.

The patients were inquired about the adverse effects of metformin which mainly included nausea, diarrhoea, dyspepsia or abdominal pain on each visit. GIT intolerance was said to be present if any one of these symptoms appeared one week after therapy with metformin.^15^ BMI before and after three months of metformin therapy was recorded.

### Data Analysis

Baseline median of the A1C and BMI of the patients was recorded along with the A1C after three months’ treatment as the data was not normally distributed. Independent t-testing was done to calculate the differences in the mean of the A1C and BMI within the groups. The frequency of GIT intolerance was calculated in overall cases and then in the response groups. Association of GIT intolerance and the cases was measured using chi-square test (p value 0.5%, x^2^=0.436).

## RESULTS

Out of 200, 119 patients were Responders whereas, 81 were found to be Non-Responders. The patients with A1C decrease of ≥0.8% were grouped as Responders and those with lesser reduction of <8% were grouped as Non-Responders to metformin therapy.

The median age of the patients was 49 years whereas in Responders it was 50 years and in Non-Responders it was 49 years. More females 69% (n=138) were affected in the present study as compared to males 31% (n=62). Amongst all the females, 88 (64%) were Responders and 50 (36%) were Non-Responders. In case of males, 56% (n=32) were Responders and 48% (n= 30) were Non-Responders. Though females were more in the study group but the gender distribution was not different in the two response groups significantly. Study showed that 54% patients had positive family history for diabetes whereas 36% patients had no family history of diabetes.

**Fig.1:**
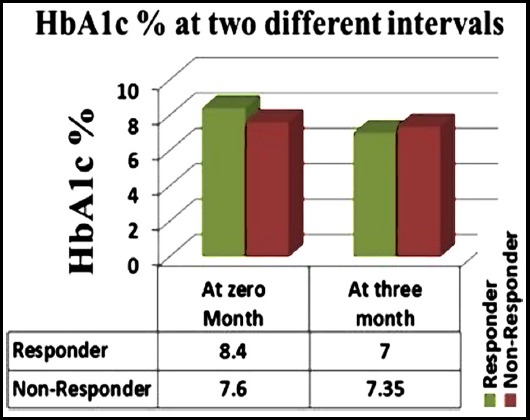
Median value of A1C at the start and after three months of Metformin therapy in Responders (N=119) and Non-Responders (N=81).

The median value of baseline A1C in Responders and Non-Responders was 8.4 and 7.6 respectively (Fig.1). The difference in the median of the two groups at the start of the metformin therapy was significant. The median value of A1C after three months of metformin therapy was seven and 7.35 in Responders in Non-Responders, respectively and the difference in the median of the two response groups was statistically significant.

In the present study the efficiency of metformin in reducing the A1C was analyzed. Group-1 included the patients with the A1C<8% and the Group-2 included patients with relatively A1C of ≥ 8%. The difference between the mean reductions in the A1C within each group was found to be statistically significant (p value <0.0001). The more the baseline A1C, the more was the decrease in A1C by metformin. The mean value of A1C in Group-1 and Group-2 at the start of the metformin therapy and three months after and the difference in the mean values of the two groups are given in Table-I.

**Table-I:** Differences in HbA1C (%) in Group-1 and Group-2 (N=200)

A1C (%)	Group-1* (Mean ± SEM)	Group-2* (Mean ± SEM)
Baseline	7.50±0.030	8.70±0.033
After 3 months	6.88±0.077	7.57±0.08
Decrease in A1C within a group	0.61± 0.07	1.13±0.08
Difference between two groups	0.51 ± 0.11	
p-value	<0.0001*	

* Group-1= Individuals with A1C ≤8%,

* Group-2= Individuals with A1C >8%

* p-value significant <0.05.

The median BMI of the diabetic patients at the start of metformin therapy was 26.09 kg/m^2^ whereas the median BMI after three months’ metformin therapy was 25.40 kg/m^2^. The difference between the median BMI before and after the metformin therapy was statistically different with *p-value* 0.00 showing the reduction of BMI after treatment. The BMI between the two groups was not statistically different showing that metformin lowering activity of BMI was same for the two response groups (Table-II).

**Table-II:** The mean and SEM of BMI before and after metformin therapy and the difference in two response groups and within the group (N=200).

BMI kg/m^2^	Responders (Mean±SEM) (n=81)	Non-Responders (Mean±SEM) (n=119)
Baseline	24.87±0.44kg/m^2^	25.24±0.55kg/m^2^
After 3 months	24.63±0.39 kg/m^2^	24.85±0.50kg/m^2^
Decrease in BMI within a group	0.24±0.11 kg/m^2^	0.39±0.20 kg/m^2^
p-value for decrease	0.00*	0.00*
Difference between two groups	0.15±0.04 kg/m^2^	
p-value for difference	0.68	

* p-value significant <0.05

**Fig.2:**
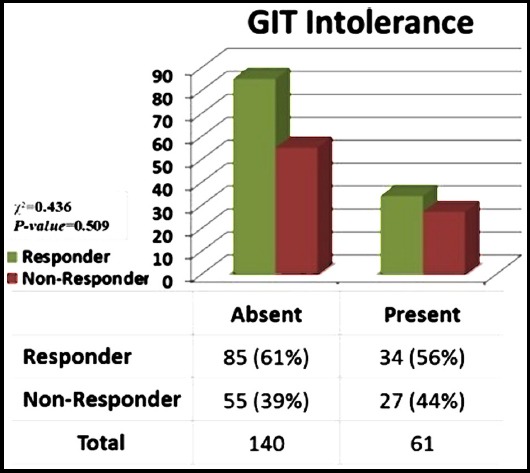
The frequency of GIT intolerance in Responders (N=119) and Non-Responders (N=81) and the association of the two variables.

After receiving metformin, about 61 patients out of 200 reported GI side effects; the difference between responders (n=34, 56%) and non-responders (n=27, 44%) was not statistically significant (*P-value* = 0.509) (Fig.2).

## DISCUSSION

Type-2 diabetes mellitus is the most prevalent form of diabetes and metformin is the first line drug for its treatment. Regardless of its widespread use, 35% of patients fail to reach initial target glycaemic control with metformin due to variable drug response.^16^ In the present study, 59.5% patients were categorized as Responders and 40.5% as Non-Responders to metformin on the basis of reductions in A1C. If categorization of patients is compared in terms of response, similar study was done on South Indian newly diagnosed T2DM patients where 76% of the patients were labeled as Responders and 23% as Non-Responders.^8^ However, the proportion of Non-responders was greater in our study but this variability in non-responsiveness may be attributed to other factors like genetic changes, duration of diabetes, or compliance of patients which were not taken in account in this study.

In the present study it was found that metformin effectively reduced the BMI of the patients after three months of therapy and these were statistically significant results which were not due to chance. The reduction in the response group was also significant but the decrement was not different amongst the two response groups. This finding was in concordance to the previous research done on white Americans as they found strong association of BMI with the patients. They found that reduction in BMI was more in Responder group as compared to Non-Responder group.^17^ Another study done on German population in 2013 also showed discordant results to the present study.^18^ The same results were found in a research done on the Australian population in 2006 and they found that metformin therapy had no effect in reducing BMI of the patient whether they are Responders or Non-Responders.^19^

Tarasova and her colleagues found that BMI was not associated to any of the response group in Latvian population and the BMI was higher than the present study. However, more attention should be paid towards classifying patients according to the degree of obesity in proportion to total body mass, as well as to the degree of abdominal obesity.^20^

GIT intolerance is one of the major side effects of metformin which leads to premature discontinuation of therapy in 4-10% of cases.^20^ The results of current study showed that GIT intolerance was not associated with the response group and these results are in contradiction to the study done on population from North Caucasia, North Africa and Sub-Sahara African ancestry. They found significant effect of metformin therapy in producing GIT intolerance.^21^ Whereas, Laura and her colleagues found no association between metformin therapy and the GIT intolerance.^22^ The risk factors for metformin intolerance should be identified like the concomitant use of anti-diarrheal drugs or the patients who have pre-existing GI problems.

Patients with initial HbA1C levels equal or more than 8% showed more decrease in these levels than those having HbA1C less than 8%. This study supports the finding of a meta-regression analysis providing a numerical estimate of an effect that has been commented on by previous authors: higher baseline A1C levels are associated with greater declines in HbA1C with metformin therapy and this is also supported by other studies.^23^

### Limitations of the study

It included using simple questioning techniques rather than validated quality of life measures to record GI adverse effects and patients’ satisfaction; not evaluating GI side effects after continued therapy; if it can improve GI compliance or not. The patients were enrolled from only one diabetic center hence limiting the diversification of the patients.

## CONCLUSION

This study revealed that, decrease in HbA1c levels by more than 0.8% from baseline could be considered a criterion for response to metformin. In addition, few patients were non-responders to metformin therapy, which may be because of combined effects of various gene polymorphisms in their interaction with non-genetic factors. Metformin reduced the BMI in all the patients; however, BMI lowering activity of metformin was same regardless of its effect on HbA1C. Moreover, the signs and symptoms of GIT intolerance did not differ between the two groups.
